# The Death-Rate of Pneumonia

**Published:** 1892-07-09

**Authors:** 


### The Practitioner's Mirror and Retrospect.

[*The Editor will be glad to receive offers of co-operation and contributions from members of the profession. All letters should be addressed to* The Editor, The Lodge, Porchester Square, London, W.]

### MEDICINE.

### THE DEATH-RATE OF PNEUMONIA.

Medical literature has been flooded with discussions on the treatment of pneumonia for seventy-five years, that is, since Laennec, by the introduction of the stethoscope, rendered its diagnosis a certainty. One method after another has been tried and discarded. Heroic and, on the other hand, purely expectant treatment, hot applications and ice, alcoholic and teetotal management, have each been advocated to the exclusion of other methods. Statistics have been accumulated to an alarming extent, and some persons think this is the chief result we have gained. Though a large proportion of them are useless and misleading, and the nature of the subject matter lends itself to every possible fallacy, yet it would seem that a few facts are firmly established, and with statistics kept in a more uniform and guarded manner, greater progress may be made. The questions involved appear at first sight few and easy to answer. Here is a disease which runs a short and definite course with few complications, but with a great mortality, generally from failure of the heart, which carries off about 1·5 per thousand of the population in temperate climates. We have, probably, no means of prevention, or of cutting short the disease, but we do require to know how we can best help our patients through and lessen the mortality. In some recent statistics, such as those of Dr. Fenwick, we have, perhaps, a firm basis to go upon in dististinguishing the effects of the many competing methods of treatment, but older records are not without value. Dr. Boardman Reed and others have compared those tabulated from the time of Laennec. At his date, and till about 1856, the method of heroic treatment by bleeding, purging, and antimony was in vogue. Laennec himself was wont to bleed once only, and that early in the course of the disease, but he used much tartar emetic, and daily bleedings were the fashion. Now Dr. Reed points out the remarkable fact that as far as we can depend on the early statistics, the mortality at the period up to 1856 was, if anything, less than in the later times of expectant or tonic treatment. Laennec himself claims that he only lost 9 per cent., and estimates the ordinary mortality at from 12 to 16 per cent. Skoda, in 1841, confirms the view that 12 per cent. was the average rate. Dr. Reed has taken the records of 6,150 cases in civil practice previous to 1856, and finds the rate to be 15·3, while among 11,100 in military hospitals the rate was actually 7·3.

After the expectant method of treatment was introduced, we should look for an improvement on these figures, but the first statistics from Vienna under the new plan give 24 per cent. Wunderlich found that he lost 23 per cent., and this rate has been fairly maintained since. Thus Reed finds that several American hospitals in recent years return their mortality as 20 to 23, 28, 30, 34, and 37 per cent., while he credits one New York hospital with the enormous rate of 48 per cent. Some hundreds of cases tabulated by collective investigation about the year 1858, many of them treated by bleeding, showed a death-rate of 9 per cent., and while those obtained by some recent American collective reports gave 13 per cent. It would be unfair to contrast these with hospital cases, nor need we enter into all the deficiences of collective investigation, except to remark that unless the earlier reporters curiously selected their cases there is no trace of improvement under recent methods. Again, if we consider that military statistics are more reliable, he contrasts the former death-rate of 7·3 per cent. in the American army with that of 61,200 cases in later years, when the rate reached 24 per cent. If the Confederate troops were added on this would be much greater, but it is only reasonable to keep them out of the calculation from the imperfect treatment they could obtain in their struggle. The earlier group of statistics embraced cases which occurred in the Mexican War, so that we are not here contrasting the results in times of peace with those in the great civil war. It is not easy to see how all these rates depend on less perfect diagnosis in earlier years. If lobular pneumonia was often formerly described as lobar, the death rate ought to have been higher instead of lower. Has the type of disease, then, been growing more severe, so as to neutralise all improvements in treatment? The English collective report in 1883 showed a mortality of 19 per cent. on 350 cases, and of those patients who were under 30 years of age one of 9 per cent., while Grisolle formerly estimated the rate for the same age at 7 per cent. It has been supposed that modern calculations include a greater number of aged persons, but we see no proof that recent mortality in early life is less than in Grisolle's cases, or in the American army rate of 7·3 or in the old English army and navy rate of 7·7.

A curious series of 1,000 cases in the Massachusetts Hospital, from 1832 to 1889, has just been quoted by Dr. Osler as showing an improvement, but the facts are the other way. The total rate steadily increased from 10 per cent. in the first decade, to 28 per cent. in the last; but this, it is argued, was due to an increase of the average age, of complicated cases, and of those in drunkards and foreigners. With a due allowance for these, the editors of the report still get a recent mortality slightly above the original 10 per cent., and, we may add, that since the earlier records are briefer, it is hardly fair to deduct those records where complications are recorded, or to suppose a great increase of intemperate cases. Anyhow, the heroic treatment compared very favourably with later methods here. The death-rate has certainly not fallen.

Dr. Fenwick last year gave us an analysis of 502 cases in the London Hospital, with a total mortality of 23 per cent., the chief point of interest being the low mortality in cases treated by ice bags and cradles, cold packs, and sponging. How far the cold treatment, and that by inhalation by oxygen, will eventually prove efficacious, remains to be seen. In the German army the death rate is said to be reduced to 8 per cent. under the use of the cold bath. But, with this exception, if we review the past, making due allowance for the differences of age, climate, and race, it seems hard to find much to choose between the results of modern methods and those of the old bleeding and purgative treatment. We do, however, recognise that the danger lies in cardiac failure, whether from high temperature, or more probably from the effects of a toxine, and that success depends on combatting this at the right time.

